# The INHABIT (synergIstic effect of aNtHocyAnin and proBIoTics in) Inflammatory Bowel Disease trial: a study protocol for a double-blind, randomised, controlled, multi-arm trial

**DOI:** 10.1017/jns.2023.113

**Published:** 2024-01-08

**Authors:** Denelle Cosier, Kelly Lambert, Marijka Batterham, Martina Sanderson-Smith, Kylie J Mansfield, Karen Charlton

**Affiliations:** 1 School of Medicine, Indigenous and Health Sciences, University of Wollongong, Wollongong, NSW, Australia; 2 Statistical Consulting Centre, National Institute for Applied Statistical Research Australia, University of Wollongong, Wollongong, NSW, Australia; 3 School of Chemistry and Molecular Bioscience and Molecular Horizons, University of Wollongong, Wollongong, NSW, Australia; 4 Graduate School of Medicine, University of Wollongong, Wollongong, NSW, Australia

**Keywords:** Anthocyanins, Clinical trial, Gut microbiota, Inflammation, Inflammatory Bowel Disease, Probiotics, Polyphenols, Randomised controlled trial, Ulcerative Colitis

## Abstract

Ulcerative Colitis (UC), a type of Inflammatory Bowel Disease (IBD), is a chronic, relapsing gastrointestinal condition with increasing global prevalence. The gut microbiome profile of people living with UC differs from healthy controls and this may play a role in the pathogenesis and clinical management of UC. Probiotics have been shown to induce remission in UC; however, their impact on the gut microbiome and inflammation is less clear. Anthocyanins, a flavonoid subclass, have shown anti-inflammatory and microbiota-modulating properties; however, this evidence is largely preclinical. To explore the combined effect and clinical significance of anthocyanins and a multi-strain probiotic, a 3-month randomised controlled trial will be conducted in 100 adults with UC. Participants will be randomly assigned to one of four groups: anthocyanins (blackcurrant powder) + placebo probiotic, probiotic + placebo fruit powder, anthocyanin + probiotic, or double placebo. The primary outcome is a clinically significant change in the health-related quality-of-life measured with the Inflammatory Bowel Disease Questionnaire-32. Secondary outcomes include shotgun metagenomic sequencing of the faecal microbiota, faecal calprotectin, symptom severity, and mood and cognitive tests. This research will identify the role of adjuvant anti-inflammatory dietary treatments in adults with UC and elucidate the relationship between the gut microbiome and inflammatory biomarkers in this disease, to help identify targeted individualised microbial therapies. ANZCTR registration ACTRN12623000630617.

## Introduction

Inflammatory Bowel Disease (IBD) is a chronic relapsing gastrointestinal disease encompassing both Crohn's Disease (CD) and Ulcerative Colitis (UC). Australia has one of the highest prevalence and incidence rates of IBD in the world, with approximately one in 250 Australians living with this disease, and an annual age-standardised incidence estimated at 29⋅3 new cases per 100 000 people.^(1,2)^ UC is characterised by inflammation in the mucosal cells of the colon, and can result in persistent gastrointestinal symptoms, fatigue, and extra-intestinal manifestations despite pharmaceutical management.^(3,4)^ Given the chronic and progressive nature of UC, the personal financial and quality-of-life costs, and the suboptimal response to medical management, effective dietary interventions that may alleviate the burden of this disease are urgently needed.^(3)^

Although the exact pathogenesis of UC is unclear, the widely accepted hypothesis involves an excessive immunological response to the intestinal microbiota and environmental factors in genetically susceptible individuals.^(5)^ Dysbiosis of the gut microbiome in people living with UC has been widely demonstrated, and is suggested to contribute to disease pathogenesis.^(6–8)^ Certain groups of beneficial gut bacteria are shown to be reduced in the gut microbiome of people with UC compared to healthy adults, particularly multiple species of butyrate-producing bacteria.^(6,9,10)^ The short-chain fatty acids (SCFAs) such as acetate, butyrate, and propionate, produced by the bacterial fermentation of non-digestible carbohydrates, have been shown to be significantly lower in adults with IBD compared to healthy controls.^(11,12)^ Of particular concern is the reduction in butyrate, which plays a significant role in maintaining intestinal barrier cells and regulating intestinal immune response.^(13,14)^ Due to difficulties in isolating and culturing butyrate-producing bacteria, they are not yet available as probiotic supplements.^(15)^ However, emerging research has identified a cross-feeding relationship between several *Bifidobacterium* and *Lactobacillus* species and butyrate-producing bacteria.^(16–19)^ Acetate, produced by *Bifidobacterium*, is a key intermediate for colonic butyrate production and acetate consumption has been commonly seen amongst butyrate-producing bacteria.^(20,21)^ Some butyrate-producing bacteria have also demonstrated the ability to utilise lactate in this cross-feeding relationship, lactate being a by-product of colonic fermentation by lactic acid bacteria including *Lactobacillus.*^(22)^ In this clinical trial, we propose supplementation with a probiotic blend containing multiple species of *Lactobacillus* and *Bifidobacterium* that have demonstrated anti-inflammatory benefits and result in increased production of butyrate SCFA.^(23–26)^

Probiotic supplementation has been shown to induce remission and reduce clinical disease activity in populations with UC. A recent systematic review and meta-analysis synthesising data from 37 IBD trials has found that probiotic supplements containing 2–3 (or more) different strains of *Lactobacillus* or *Bifidobacterium*, and administered at a daily dose of 10^9^–10^12^ colony-forming unit (CFU)/day were able to induce disease remission, particularly in UC, and proliferate *Bifidobacterium* and *Lactobacillus* in the gut microbiota of participants.^(27)^ Another recently published systematic review and meta-analysis found that, of 25 studies amongst adults with UC, 21 showed probiotics to be effective in achieving or maintaining remission. Supplementation with *Bifidobacterium sp.* or a combination of probiotics was the most effective intervention compared to control.^(28)^

It is established that anthocyanins, a polyphenol subclass found in red-, blue- and purple-coloured fruits and vegetables,^(29)^ exert anti-inflammatory effects and interact with the gut microbiome, and therefore may be beneficial adjuvant treatments in UC.^(30)^ There are multiple preclinical studies supporting the anti-inflammatory benefits of anthocyanin supplementation in populations with UC.^(31)^ This evidence is largely obtained from animal and laboratory models, and therefore further clinical trials are necessary to provide translational evidence. Evidence from animal and *in vitro* studies suggests that polyphenols, including anthocyanins, function as prebiotics in the gut microbiota and can stimulate the growth of beneficial microbes including those shown to be lacking in adults with UC.^(32–35)^ The increase in beneficial gut microbes and consequent production of metabolites, including SCFAs, has been shown to reduce inflammatory biomarkers.^(34,35)^ What remains unclear is whether and how probiotic supplementation enriched with anthocyanins influences disease activity, inflammation and the gut microbiome in UC.

We have identified a significant gap in the literature pertaining to a lack of clinical trials and data that test if there are changes to both inflammatory markers and the gut microbiota profile after supplementation with microbiota-modulating therapies in UC. Both polyphenols and probiotics have been shown to reduce inflammation, however the magnitude may not be clinically significant in this population.^(36–38)^ Thus, we aim to test, for the first time, whether the combination of these two separate interventions results in synergistic effects with a greater magnitude of change to intestinal inflammation and therefore beneficial disease control. We additionally aim to assess whether changes in inflammation and the gut microbiota are associated with an improved quality of life and a reduction in symptom burden.

### Research aim and objectives

This study aims to evaluate the combined effect and clinical significance of a multi-strain probiotic intervention (VSL#3®^,^ containing one strain of *Streptococcus thermophilus*, three strains of *Bifidobacterium* and four strains of *Lactobacillus*) provided together with dietary anthocyanins extracted from New Zealand-grown blackcurrants on the gut microbiota profile and inflammatory biomarkers in adults with UC.

We hypothesise that (1) anthocyanins, in combination with probiotics, will result in a reduction in gut inflammation measured by faecal calprotectin (FCAL) and will modulate the gut microbiome measured through shotgun metagenomic sequencing of faecal samples; (2) anthocyanins, in combination with probiotics, will result in a synergistic effect that results in a significantly greater reduction in FCAL and improvement in the gut microbiome than each intervention alone; (3) the reduction in FCAL will be explained by beneficial changes to the gut microbiome profile; and (4) these changes in FCAL and the gut microbiome will lead to clinically significant improvements in health-related quality-of-life measured with the IBDQ-32.

## Methods

This is a double-blind randomised controlled trial protocol for examining the effectiveness of anthocyanins alone, and in combination with a multi-strain probiotic, over 3 months for adults aged ≥18 years with UC. One hundred eligible participants will be randomly assigned to one of four intervention arms in a 1:1:1:1 ratio for a 3-month duration: (1) 310 mg of anthocyanin derived from New Zealand blackcurrants with a placebo probiotic; (2) multi-strain probiotic with a placebo fruit powder; (3) 310 mg anthocyanin plus a multi-strain probiotic; (4) double placebo. Written informed consent will be obtained from all participants prior to commencing the trial.

### Recruitment

Participants will be recruited from across Australia, utilising gastroenterology and research networks. Additionally, social media advertising will be utilised to allow interested participants to contact the co-ordinating researcher. Recruitment will remain open until the sample size is met.

#### Sample size and power calculations

Our sample size (*n* 100) was calculated in G*Power Version 3.1.9.6 based on the primary outcome measure of a clinically relevant change in mean score of ≥16 in the 32-item Inflammatory Bowel Disease Questionnaire (IBDQ-32).^(39,40)^ The primary comparison of interest is between the combined intervention versus control. Assuming a mean change in the IBDQ-32 score of 16 ± 20 estimated from previous literature,^(41–44)^ α- significance of 0⋅05 and power 95 %, a sample size of n 19 per arm is required with effect size of 0⋅8. To allow for ~25 % drop-out, given the duration of this trial and based on previous literature,^(45–47)^ *n* 25 participants will be recruited to each arm.

### Inclusion and exclusion criteria

Inclusion criteria: (1) diagnosis of UC for at least 3 months prior to screening, documented by endoscopic and histologic findings; (2) aged ≥18 years; (3) willing and able to take oral supplements; (4) able to communicate in the English language.

Exclusion criteria: (1) CD; (2) change in antibiotics or probiotics in past 3 months, including commencing antibiotics/probiotics, ceasing antibiotics/probiotics or changing the type of antibiotic/probiotic consumed; (3) escalation to biologics or immunomodulators in the past 6 weeks; (4) currently taking steroids >20 mg daily; (5) bowel resection surgery; (6) pregnant and lactating women; (7) end-stage liver or kidney failure; (8) current treatment for *Clostridium difficile* or other intestinal infection; (9) sucrose-isomaltose deficiency or maltose intolerance.

As per the Medical Advisor's recommendation, all participants must have stable medication for 2 weeks prior to commencing the study. All types of IBD medications are permitted in this study. Participants who have taken antibiotics or probiotics consistently for 3 months prior to recruitment will be permitted to commence the study, provided they agree to continue taking the probiotic/antibiotics throughout the study. Patients receiving topical therapy (enemas or suppositories) are eligible to participate if they are willing to continue the therapy at a stable dose throughout the study. Participant receiving steroids >20 g/day can commence the study 2 weeks after their dosage has reduced to ≤20 mg/day. Participants treated with steroids ≤20 mg/day are eligible to participate if they plan to maintain this stable dose for all the study duration.

Participants who require a change in their medication treatment related to uncontrolled UC activity (i.e. increase in steroids or mesalamine doses, start of steroids or biologics or immunosuppressants) during the 12-week intervention, those who do not comply with the study protocol, and those who voluntarily leave the study will not be included in the per-protocol analyses. These participants will be encouraged to continue to complete required outcome data and attend the final visit for inclusion in the intention-to-treat (ITT) analysis.

### Screening procedure

All interested participants will complete a screening survey covering all aspects of the inclusion and exclusion criteria. If eligible, participants will be contacted by the researchers and sent a participant information sheet and consent form. A baseline appointment will then be scheduled.

### Randomisation and allocation concealment

**Fig. 1.**
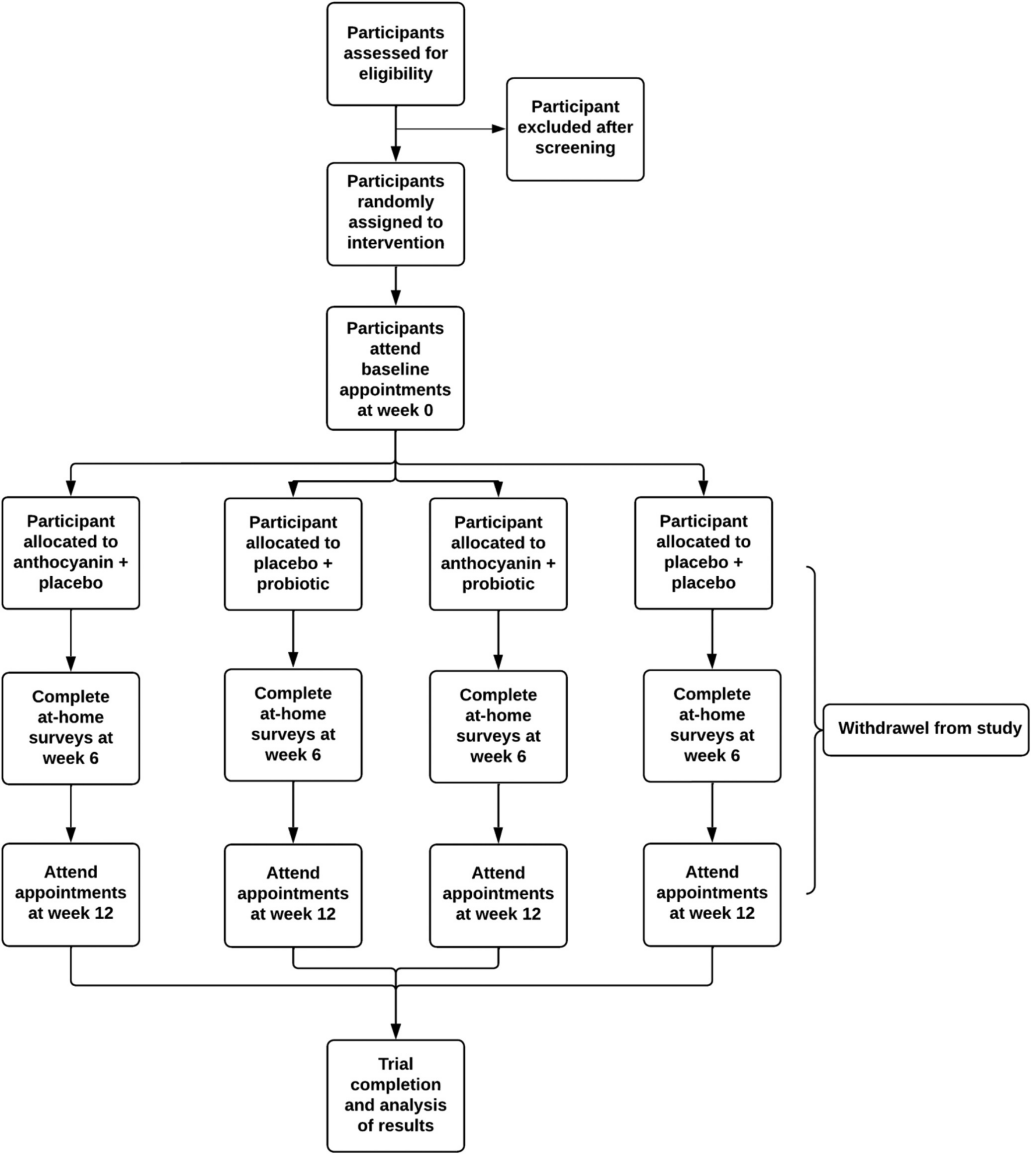
Consolidated standards of reporting trial flow diagram of the study schedule.

Participants will be block randomised upon enrolment into one of four categories by a single researcher who will not be involved in data collection, and who will organise the corresponding active or placebo products for each participant. For blinding purposes, the code allocation will be concealed and known only to this researcher until statistical analysis has been completed. In the case that unblinding is required for medical reasons or adverse reactions, the researcher responsible for randomisation will reveal the product allocation to the participant. If the participant continues in the study, their results will be included in the ITT analysis but excluded from per-protocol due to unblinding. A Consolidated Standards of Reporting Trials (CONSORT) flow chart outlining the study schedule is displayed in Fig. 1.

### Interventions

#### Anthocyanin intervention

12 g of freeze-dried New Zealand blackcurrant powder (Arepa Neuroberry®) provided by Arepa Holdings will be consumed daily by participants in intervention arms 1 and 2. This provides 310 mg of anthocyanins/day. The product can be mixed in any cold, low acidity, non-carbonated drink, or food (i.e. smoothie, juice, water, milk or on yoghurt or cereal). Previous research has shown no statistically significant adverse reactions or harm associated with polyphenol interventions in adults with IBD.^(37)^

#### Probiotic intervention

Participants in intervention arms 2 and 3 will receive the multi-strain probiotic VSL#3® supplied by Actial Farmaceutica, in the form of two daily sachets. This probiotic contains one strain of *Streptococcus thermophilus* BT01, three strains of Bifidobacteria (*B. breve* BB02; *B. animalis* subspecies [subsp] *lactis* BL03, previously identified as *B. longum* BL03; and *B. animalis* subsp. *lactis* BI04, previously identified as *B. infantis* BI04), and four strains of Lactobacilli (*L. acidophilus* BA05, *L. plantarum* BP06, *L. paracasei* BP07, and *L. helveticus* BD08, previously identified as *L. delbrueckii* subsp. *bulgaricus* BD08). VSL#3® contains no less than 450 billion colony-forming units (CFUs) per single sachet. The product can be mixed in any cold, low acidity, non-carbonated drink or food (i.e. smoothie, juice, water, milk, or on yoghurt or cereal). Probiotic interventions in adults with IBD have shown a risk of abdominal pain and other gastrointestinal side effects such nausea, abdominal cramping and flatulence. No other harms associated with probiotic interventions in this population group have been documented.^(48)^

#### Placebo

Participants in intervention arms 3 and 4 will receive 12 g of a placebo powdered fruit extract with texture and taste matched to the blackcurrant powder. Participants in intervention arms 1 and 4 will receive two placebo sachets of similar appearance to the provided probiotic.

### Outcome measures

See Table 1 for the timeline of outcome measures.

**Table 1.** Data collection schedule for four study arms

Outcomes	Measure/Method	1st Interview	6 weeks	2nd Interview	Early termination interview
Anthropometry	Self-reported height and weight	X		X	X
Clinical	Inflammatory Bowel Disease Questionnaire	X	X	X	X
	Inflammatory Bowel Disease Symptom Inventory – Short Form	X	X	X	X
	Bristol Stool Chart	X		X	
	Shotgun metagenomic sequencing of faecal sample	X		X	
	Faecal Calprotectin	X		X	
	Three non-consecutive 24 h dietary recall	X	X	X	
Lifestyle and medical history	Demographic survey	X			
	Medications and IBD clinical history	X			X
	SCCAI	X		X	X
Cognitive performance, mood, and attention	DASS-21 questionnaire	X		X	
	Stroop test	X		X	
	RAVLT	X		X	

#### Health-related quality of life (HRQOL)

The primary outcome of HRQOL will be measured using the IBDQ-32. The IBDQ-32 is a validated 32-item self-reported questionnaire, assessing quality of life (QOL) across four disease-specific sub-categories: bowel symptoms (ten questions); systemic symptoms including sleep disorders and fatigue (five questions); emotional function (twelve questions); and social function (five questions).^(49)^ The total score ranges from 32 to 224 points, with lower scores reflecting lower HRQOL. Each question is scored using a 7-point Likert scale ranging from 1 (low QOL) to 7 (high QOL). All participants will complete the IBDQ-32 at baseline, 6 weeks, and 12 weeks. The change in total IBDQ-32 score has been demonstrated to detect, with reliability and sensitivity, changes in disease activity and endoscopic mucosal healing in response to treatment.^(40,50,51)^ A change in total score of ≥16 points has been shown to correlate to a clinically meaningful change in QOL.^(39,40)^

#### Secondary outcome measures

Shotgun metagenomic sequencing of faecal microbiota samples will be performed at baseline and 12 weeks, to profile the taxonomic composition and functional potential of all microbial communities present in the faecal microbiota.^(52)^ This sequencing will capture any changes to the composition and functional potential of the faecal microbiota after the intervention.

Faecal samples collected for shotgun metagenomic sequencing will be collected in commercial faecal kits from Microba Life Sciences. The Microba commercial kit includes a Copan FLOQSwab in an active drying tube that has been tested to preserve samples at room temperature for up to 10 weeks. Samples will be sent via post to Microba Life Sciences and will be stored at −80 °C until all samples are collected. Metagenomic sample processing and sequencing will be performed at Microba Life Sciences. Faecal samples will be extracted using the DNeasy 96 PowerSoil Pro QIAcube HT Kit (Qiagen 47021) in 2-ml deep well plate format as per manufacturer's instructions with a modified initial processing step on the QIAcube HT DNA extraction system (Qiagen 9001793). Libraries will be constructed using the Illumina DNA Prep (M) Tagmentation Kit (Illumina, 20018705) with IDT for Illumina DNA/RNA UD Index Sets A-D (Illumina 20027213-16) according to manufacturer's instructions, with a modification of volume to accommodate processing in a 384-plate format. Resulting libraries will be assessed using a high sensitivity dsDNA fluorometric assay (QuantIT, ThermoFisher, Q33120) and individual libraries are visualised with capillary gel electrophoresis using the QIAxcel DNA High Resolution Kit (Qiagen, 929002). Individual libraries will be pooled in equimolar amounts to create a sequencing pool which is assessed using a high sensitivity dsDNA fluorometric assay (QuantIT, ThermoFisher, Q33120) and will be visualised with capillary gel electrophoresis using the QIAxcel DNA High Resolution Kit (Qiagen, 929002). Sequencing pools will then be loaded and sequenced on the NovaSeq6000 (Illumina) using v1.5 300 bp PE sequencing reagents, according to the manufacturer's instructions. Metagenomic sequencing data quality control will be performed at Microba Life Sciences. Paired-end DNA sequencing data will be demultiplexed and adaptor trimmed using Illumina BaseSpace Bcl2fastq2 (v2.20) accepting one mismatch in index sequences. Reads will then be quality trimmed and residual adaptors removed using the software Trimmomatic v0.39^(53)^ with the following parameters: -phred33 LEADING:3 TRAILING:3 SLIDINGWINDOW:4:15 CROP:100000 HEADCROP:0 MINLEN:100. All samples will then be randomly subsampled to a standard depth of seven million read pairs.

#### Faecal calprotectin

FCAL will be measured at baseline and 12 weeks. FCAL is a sensitive marker of intestinal inflammation and has been shown to correlate to histological and endoscopic inflammation and disease activity measures.^(54,55)^

Faecal samples for FCAL will be collected in an ELISA kit (Epitope Diagnostic) and stored at −80 °C until all samples are collected. FCAL will be determined with a range of 0–321 μg/g or 0–892 ng/ml calprotectin. The analytical sensitivity (LLOD) of the human calprotectin ELISA as determined by the 95 % confidence limit on 12 duplicate determination of zero standard is approximately 2⋅5 ng/ml. A LLOQ was determined by dilution of assay standards and it is approximately 5 ng/ml.

#### Gastrointestinal symptoms

Gastrointestinal symptoms will be assessed via the IBD Symptom Inventory-Short Form (IBDSI-SF)^(56)^ and stool consistency self-rated against Bristol Stool chart. These outcomes will be assessed at baseline, 6 weeks, and 12 weeks, to detect any beneficial or adverse changes due to the intervention.

Cognition, memory and mood will be assessed at baseline and 12 weeks via the Stroop test, Rey Auditory Verbal Learning Task (RAVLT) and DASS-21 questionnaire.

#### Data collection procedures

Two weeks prior to study commencement, participants will receive faecal sample testing kits by mail with instructions for collection one day prior to their baseline online appointment. Participants will attend a baseline online meeting for completion of a demographic and health surveys, completion of the cognitive performance, mood and attention tests, and instructions regarding ongoing study assessments. Participants will return completed faecal samples by express post and will receive intervention products. Participants will be instructed to avoid making dietary changes throughout the intervention period and refrain from introducing new dietary products or supplements containing probiotics or prebiotics. Brief education on these products will be provided by an Accredited Practising Dietitian. Dietary intake will be captured by three 24 h dietary recalls completed on non-consecutive days at baseline, 6 and 12 weeks. Participants will be required to recall all food and drink items consumed in the previous 24 h period on two weekdays and one weekend day. This will be completed using the online Intake24 multiple-pass dietary recall system,^(57–59)^ in which all foods and drinks are coded to the AUSNUT 2011–13 database.^(60)^ Dietary recalls will be collected using REDCap electronic data collection tools,^(61,62)^ with summaries of intake then imported by the researchers into FoodWorks Professional nutrient analysis software.^(63)^ Dietary intake will also be analysed for dietary polyphenol content using Phenol-Explorer database.^(64)^ Participants will be instructed to remain on required medical therapy throughout the intervention. Demographic and health surveys will be delivered online to participants via REDCap.

To improve participant adherence, the participants will receive email and SMS contact in advance of every visit and mid-way assessments. Telephone contact will be made if no replies through email or SMS are received. If participants fail to attend treatment sessions, researchers will enquire as to the reasons for non-attendance and aim to encourage adherence to treatment and attendance at sessions via telephone contact. Participants will be provided with a checklist and requested to complete it each day indicating whether they consumed the study products. As an additional marker of compliance, participants will be required to inform the researchers of any remaining products at the end of the intervention. Participants will also be asked a question in the dietary assessment Intake 24 instrument about their consumption of the provided supplements on the previous day. Affirmative responses will be summed across the 3 days for each of the time points at which the dietary assessment is conducted (for example, if participants report taking the supplements on only one of the 3 days this will indicate 33 % compliance).

### Statistical analysis

Statistical analyses will be performed in Stata software,^(65)^ R software^(66)^ and/or IBM SPSS Statistics.^(67)^ An ITT analysis will be the primary analysis, including all participants randomised and analysed according to the treatment group to which they were originally assigned. A per-protocol analysis will additionally be conducted for all outcomes and used as a sensitivity analysis compared to the ITT. The per-protocol analysis will include participants who completed the trial without major violation including medical disruptions or unblinding, and who exhibited >80 % compliance to the intervention products.

Baseline demographics and measurements will be reported for each intervention group as means and standard deviations or 95 % CI.

The primary outcome will be the mean IBDQ-32 score at week 12 adjusted for baseline and covariates and assessed using an ANCOVA. A sensitivity analysis of differences in within-group change in IBDQ-32 score from baseline to week 12 will be measured using a two-sample *t*-test or Mann–Whitney *U*-test depending on normality of distribution. Within-group differences in secondary outcomes measured at two timepoints will be evaluated using a two-sample *t*-test or Mann–Whitney *U*-test depending on normality of distribution. Within-group differences in secondary outcomes measured at three time points will be evaluated using linear mixed modelling. Between-group differences of outcomes will be explored using ANOVA or ANCOVA if covariates need to be considered or mixed models. Relevant covariates associated with the primary outcome of health-related quality-of- life will be included in the analyses. Secondary analyses will be conducted to investigate relationships between changes in outcome measures. Predictors and assumptions of missing data will be investigated. Methods such as multiple imputation will be considered depending on the data missing. Appropriate sensitivity analyses will be performed.

### Data management

After obtaining informed consent from participants, the collected data will be stored in secure electronic databases within the University of Wollongong network by the trial coordinator (DC), accessible only to the specified members of the research team. Once participants are enrolled in the study and assigned a participant ID, participant names will be de-identified from documents and replaced with participant ID. All data will be stored electronically for 15 years before destruction. The final trial dataset will only be accessible to the specified members of the research team. A data safety monitoring board will not be convened for this clinical trial due to the absence of significant risk associated with the interventions and the early stage of this clinical trial.^(68)^ The research team will meet fortnightly during the conduct of the trial to oversee data collection and storage procedures.

## Discussion

To our knowledge, the current trial will be the first to investigate the combined effect of an anthocyanin and a multi-strain probiotic as an adjuvant therapy in mild-to-moderate UC. The results of the analysis may provide insights into the relationship between reduction of intestinal inflammation and changes to the faecal microbiota, which thus far have been studied separately in clinical trials of adults living with UC. This data may contribute towards the pursuit of targeted and individualised microbial therapies for treatment of inflammation and dysbiosis in adults living with UC. The findings of this study will additionally expand the current knowledge on the role of anthocyanins and specific probiotic strains as anti-inflammatory therapies in UC and may contribute towards the development of adjuvant anti-inflammatory dietary therapies that improve clinical outcomes, increase the QOL, and alleviate the burden of this disease for people living with IBD.
